# Xylem sap residue in cut-open conduits can affect gas discharge in pneumatic experiments

**DOI:** 10.1093/treephys/tpae092

**Published:** 2024-07-24

**Authors:** Marcela T Miranda, Luciano Pereira, Gabriel S Pires, Xinyi Guan, Luciano M Silva, Swetlana Kreinert, Eduardo C Machado, Steven Jansen, Rafael V Ribeiro

**Affiliations:** Laboratory of Plant Physiology ‘Coaracy M. Franco’, Center for Agricultural and Post-Harvest Biosystems, Agronomic Institute (IAC), Av. Dr. Theodureto de Almeida Camargo 1500, 13075-630 Campinas, SP, Brazil; Institute of Botany, Ulm University, Albert-Einstein-Allee 11, 89081 Ulm, Germany; Institute of Botany, Ulm University, Albert-Einstein-Allee 11, 89081 Ulm, Germany; Laboratory of Crop Physiology (LCroP), Department of Plant Biology, Institute of Biology, University of Campinas (UNICAMP), Rua Monteiro Lobato 255, 13083-862, Campinas, SP, Brazil; Institute of Botany, Ulm University, Albert-Einstein-Allee 11, 89081 Ulm, Germany; Institute of Botany, Ulm University, Albert-Einstein-Allee 11, 89081 Ulm, Germany; Institute of Botany, Ulm University, Albert-Einstein-Allee 11, 89081 Ulm, Germany; Laboratory of Plant Physiology ‘Coaracy M. Franco’, Center for Agricultural and Post-Harvest Biosystems, Agronomic Institute (IAC), Av. Dr. Theodureto de Almeida Camargo 1500, 13075-630 Campinas, SP, Brazil; Laboratory of Crop Physiology (LCroP), Department of Plant Biology, Institute of Biology, University of Campinas (UNICAMP), Rua Monteiro Lobato 255, 13083-862, Campinas, SP, Brazil; Institute of Botany, Ulm University, Albert-Einstein-Allee 11, 89081 Ulm, Germany; Laboratory of Crop Physiology (LCroP), Department of Plant Biology, Institute of Biology, University of Campinas (UNICAMP), Rua Monteiro Lobato 255, 13083-862, Campinas, SP, Brazil

**Keywords:** artifact, embolism, Pneumatron, vulnerability curves

## Abstract

Considerable attention has been paid to addressing methodological concerns related to measurements of embolism in conduits of angiosperm xylem. A fast, easy and cheap method is based on gas extraction measurements from dehydrating samples to obtain pneumatic vulnerability curves (VCs). Here, we tested the assumption that cutting open conduits leads to gas-filled lumina when these are cut in air at fairly high water potentials, which is required to detect embolism in intact conduits. We performed VCs with the Pneumatron for 12 angiosperm species and extracted sap from cut-open vessels in branches of nine species under early stages of branch dehydration. The optical method was applied to *Citrus* plants as an alternative reference method to estimate embolism resistance. We found an increase in gas discharge during early stages of dehydration, which affected the pneumatic VCs for most of the species studied. Xylem sap residue was not absorbed immediately by surrounding tissue in cut-open conduits in six of the nine species but gradually disappeared over time during progressive dehydration. The amount of gas discharged increased until all residual sap was absorbed, and was not related to embolism. We conclude that residual xylem sap in cut-open conduits affects early stages of pneumatic VCs and represents a novel artifact that can easily be corrected for. Yet, it remains unclear why exactly the air–water meniscus in cut-open conduits did not fully withdraw to the conduit end wall in most species. By analyzing the slope of VCs over time, we could improve estimations of embolism resistance, as evidenced by a strong agreement between the pneumatic and the optical methods. Since residual sap in cut-open conduits of some species could slightly underestimate embolism resistance, we propose to apply a correction for this artifact based on the high time-resolution measurements taken with a Pneumatron.

## Introduction

Xylem sap in water-conducting cells of plants is frequently under negative pressure, and therefore prone to changes in liquid–gas interfaces, which may lead to transport failure by embolism (Dixon and Joly 1895). Studies on the mechanisms of sap transport and embolism formation are hampered by the varied size dimensions of the xylem pathway, which range from micrometer-wide wide conduits to nanometer-sized pore constrictions in pit membranes and cell walls. Additionally, the dynamic complexity of multiphase interactions among liquids, gasses and cell walls further complicates these studies (Kaack et al. 2021). Moreover, the manipulation of xylem tissue, especially when sap is under negative pressure, can result in artifacts and affect the accuracy of plant embolism evaluation (Wheeler et al. 2013; Jansen and Schenk 2015; Lamarque et al. 2018).

Embolism resistance varies among species and is typically evaluated by a branch vulnerability curve (VC), which gives the relationship between xylem water potential and the corresponding degree of xylem embolism (Pereira et al. 2016). When a plant dehydrates, its xylem water potential decreases, and the likelihood of embolism formation may increase. Three important traits related to VCs are the water potential values inducing 12%, 50% and 88% of embolism (Ψ_12_, Ψ_50_ and Ψ_88_, respectively). While Ψ_12_ is considered to be the threshold for air entry in the xylem causing embolism, Ψ_50_ is commonly used to compare the resistance of a particular species to embolism, and Ψ_88_ represents the water potential associated with the threshold leading to irreversible drought damage and mortality risk (Urli et al. 2013).

Several methods have been developed to study xylem vulnerability to embolism, which may be estimated either indirectly by measuring the loss of conductivity due to embolism formation, or directly by quantifying the gas volume of embolized vessels (Venturas et al. 2019). The loss of conductivity can be measured with a hydraulic apparatus (Sperry et al. 1988) or by the centrifuge method (Cochard 2002; Peng et al. 2019), whereas embolism can be quantified through imaging methods (Brodersen et al. 2010; Brodribb et al. 2016; Meixner et al. 2020), or gas extraction using the Pneumatic method (Pereira et al. 2016, 2020*a*).

The Pneumatic method quantifies the amount of gas that can be extracted from intact (i.e. non-cut-open) and embolized conduits that are connected via interconduit pit membranes to cut-open conduits. Increases in the discharged air volume during progressive dehydration of the branch are then related to the progressive spread of embolism in intact conduits (Pereira et al. 2016). In fact, large gas volume is extracted when embolism has been induced in intact conduits (Guan et al. 2021; Yang et al. 2023). Recently, Pneumatron devices have been used to obtain pneumatic VCs (Pereira et al. 2020*a*). These devices are automated instruments that capture at a high temporal resolution the amount of gas extracted from xylem conduits, and therefore evaluate the gas kinetics during xylem dehydration over several hours to days. Detailed modeling of gas diffusion kinetics that underlies pneumatic VCs, combined with a comparison of various methods, provide strong evidence to consider the Pneumatic method as a highly accurate, easy and low-budget method to estimate embolism resistance of xylem (Guan et al. 2021; Brum et al. 2023; Paligi et al. 2023; Yang et al. 2023).

Nevertheless, pneumatic measurements should be interpreted carefully to avoid misinterpretation of data, considering the basic assumptions associated with this method (e.g. Chen et al. 2021; Brum et al. 2023). A central assumption, for instance, is that conduits cut open under atmospheric conditions become fully gas-filled because sap in cut-open conduits would be quickly withdrawn into neighboring intact conduits due to its negative pressure (Van Ieperen et al. 2001; Tyree and Zimmermann 2002). Earlier work on pneumatic VCs of *Citrus* species, however, indicated that this assumption may not be correct and that sap may remain in cut-open conduits, affecting the amount of gas discharged (GD) in pneumatic experiments (Miranda et al. 2024). If sap remains in cut-open conduits, this may especially affect the accuracy of gas discharge measurement during initial dehydration stages and then undermine the minimum amount of GD before embolism propagation occurs. It is important that cut-open conduits in pneumatic experiments are filled with gas because these conduits are considered an extension of the discharge tube of the Pneumatron.

Here, we tested if cut-open conduits are fully filled with gas when these are cut open in air, and whether partial or complete sap removal affected estimations of embolism resistance in pneumatic experiments. Besides *Citrus* species, we aimed to examine pneumatic VCs of 11 additional angiosperm species to test if a possible artifact induced by residual sap is common. We also examined if residual sap in cut-open vessels is related to the xylem water potential, and how pneumatic VCs could be corrected for a potential artifact caused by residual xylem sap.

## Materials and methods

### Plant material

We constructed VCs using the Pneumatron for species from two sites with different climatic conditions. Except for *Citrus* and *Coffea* plants that were growing in pots, all samples from the other species were collected from mature trees. At the greenhouses of Ulm University (Germany, 48°25′20′′N, 9°57′20′′E, 620 m a.s.l.), 2-year-old plants (*n =* 4) of ‘Doppio Sanguinello’ blood orange (*Citrus sinensis* (L.) Osbeck grafted on *Poncirus trifoliata* (L.) Raf.) were grown in 5-L pots filled with commercial soil (Flora-Toskana GmbH, Kempten, Germany) and 6-year-old plants of *Coffea arabica* (L.) were grown in 18-L pots filled with the same soil. Mature trees from a forest at Ulm University were also evaluated, and samples of the following species were collected during the summer of 2021: *Acer campestre* (L.) (*n =* 6), *Acer pseudoplatanus* (L.) (*n =* 3), *Carpinus betulus* (L.) (*n =* 6), *Fagus sylvatica* (L.) (*n =* 6), *Populus tremula* (L.) (*n =* 4), *Prunus avium* (L.) (*n =* 3), *Quercus petraea* (Matt.) Liebl (*n =* 6) and *Quercus robur* (L.) (*n =* 6). At the Experimental Station of the Agronomic Institute (Campinas SP, Brazil, 22°52′18′′S, 47°04′39′′W, 679 m a.s.l.), branches of *Olea europaea* (L.) (*n =* 5) were collected from mature trees in May 2021. For *Eucalyptus camaldulensis* Dehnh (*n =* 3), we retrieved pneumatic VCs presented previously (Pereira et al. 2020a), which were based on mature trees growing in Campinas SP, Brazil.

During summer, branches of about 1-m long and 10 to 15 mm in diameter were cut from plants early in the morning and transferred to the laboratory into plastic bags, with the cut end kept under water, which took about 5 min. Branches were kept in moist plastic bags in the dark for at least 60 min to ensure that stomata were closed before the pneumatic and optical method were applied.

### Pneumatic VCs of branches

A Pneumatron apparatus was used to measure the gas diffusion kinetics of desiccating branches (Jansen et al. 2020; Pereira et al. 2020*a*; Trabi et al. 2021). Measurements were taken every 15 min, and the xylem water potential was monitored in bagged leaves with a pressure chamber (PMS 1505D, PMS Instruments, Corvalis, OR, USA). Using a vacuum pump, 40 kPa of absolute pressure was applied to a rigid tube with known volume that was connected with the proximal end of a branch to extract gas. The vacuum pump reached 40 kPa (i.e. the initial pressure Pi) within less than 1 s and pressure was recorded every 500 ms during the gas discharge phase. The final gas pressure (Pf) was taken after 15 s. According to the ideal gas law, the moles of air extracted from vessels (Δn, mol) were given as follows:


(1) \begin{equation*} \Delta n=n\mathrm{f}-n\mathrm{i}=\frac{\left(P\mathrm{f}-P\mathrm{i}\right)\times V}{RT}, \end{equation*}

where *n*i and *n*f represented the initial and final number of moles of air at the initial and final pressure, respectively. *V* was the fixed discharging tube volume (in l), *R* is the gas constant (8.314 kPa l mol^−1^ K^−1^) and *T* is the room temperature (293.15 K).

The equivalent amount of GD (in μL) at atmospheric pressure (*P*_atm_, 98 kPa) and the percentage of gas discharged (PGD, %) were calculated as


(2) \begin{equation*} \mathrm{GD}=\frac{\Delta \mathrm{n} RT}{P_{\mathrm{atm}}}\times{10}^6 \end{equation*}

(3) \begin{equation*} \mathrm{PGD}=100\times \frac{\mathrm{GD}-{\mathrm{GD}}_{\mathrm{min}}}{{\mathrm{GD}}_{\mathrm{max}}-{\mathrm{GD}}_{\mathrm{min}}}, \end{equation*}

where GD_min_ was the minimum volume of air when the branch was well-hydrated, and GD_max_ was the maximum volume when the branch was severely dehydrated and GD stopped increasing, even with decreasing xylem water potential.

The VCs were then generated by plotting PGD or the percentage of embolized pixels (PEP, %, see below for the optical method) against xylem water potential (Ψ) and by fitting the following equation (Pammenter and Vander Willigen 1998):


(4) \begin{equation*} \mathrm{PGD}\ \mathrm{or}\ \mathrm{PEP}=\frac{100}{1+\exp \left(\frac{\mathrm{S}}{25}\right)\ \left(\Psi -{\Psi}_{50}\right)}, \end{equation*}

where *S* represented the slope of the curve, and Ψ_50_ the xylem water potential corresponding to 50% of GD_max_. The xylem water potentials at 12% and 88% of GD, known as Ψ_12_ and Ψ_88_, respectively, were calculated following Domec and Gartner (2001):


(5) \begin{equation*} {\Psi}_{12}=\frac{2}{\left(\frac{\mathrm{S}}{25}\right)+{\Psi}_{50}} \end{equation*}

(6) \begin{equation*} {\Psi}_{88}=\frac{-2}{\left(\frac{\mathrm{S}}{25}\right)+{\Psi}_{50}}. \end{equation*}

### Adjustment of the pneumatic measurements

**Figure 1:**
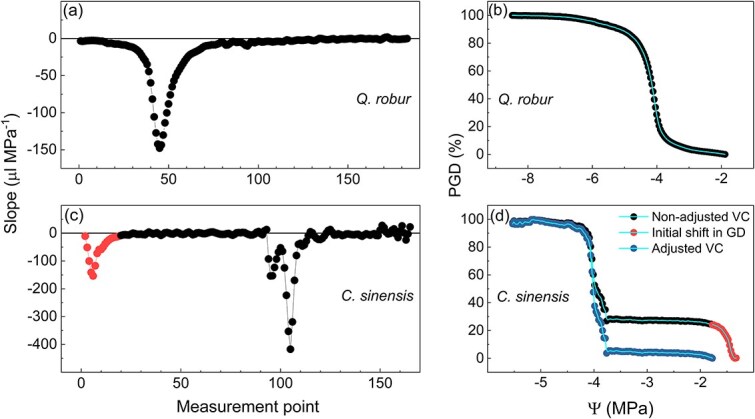
Comparison of pneumatic VCs without (*Q. robur* in a and b), and with an initial shift in gas discharge (*C. sinensis* in c and d). For the correction of the curves, a spline function was fitted at the VCs (light blue lines in b and d), and then the equation from the spline fitting was derived and the slopes plotted against the measurement point (in a and c). When there was no initial shift in the amount of GD, the slope during early stages of dehydration was close to zero, providing a minimum plateau in the VC (a), and no adjustment was performed (b). When there was an initial shift in GD, the slope at beginning of the dehydration process was far from zero, as shown by the red symbols in (c). In this case, the points corresponding to the initial shift in GD (red points in d) represented an artifact and were removed to correct the VC (blue symbols in d).

To determine the beginning of the plateau in the VCs obtained with the Pneumatron, a cubic spline interpolation was fitted for GD as function of Ψ (Fig. 1b and d). The spline interpolation used was that of Forsythe, Malcolm and Moler (Forsythe et al. 1977; R Core Team 2023). The equation from the spline fitting was derived and then the slope at each point was checked. By plotting the slope of the VCs by each measurement point over time (Fig. 1a and c), we were able to identify when there was a shift in GD at the beginning of the curve (Fig. 1d). When there was an initial shift in GD, the slope was higher at the beginning of the curve and then reached values close to zero, which represented the initial plateau, as observed for *C. sinensis* in Fig. 1c. In that case, the initial plateau was automatically identified as the smallest slope (close to zero) at the beginning of the curve, excluding the initial increase in GD. In other words, all GD measurements preceding the initial plateau were removed as the correction procedure for the artifact described in this paper. When there was no initial shift in GD, as observed for *Q. robur* in Fig. 1a, the slope of the curve at the beginning of the measurements showed constant values close to zero, and no adjustment or correction was applied (Fig. 1a and b).

### Optical measurements on leaves and branches of Citrus plants

A healthy, terminal branch of *C. sinensis* was selected to apply the optical method. This branch had a stem diameter of ca 3 to 6 mm and a length of ca 50 cm A leafless region of the branch was prepared. Bark tissue ~15 to 20 mm in length was carefully removed from one side of the branch to expose the underlying wood without causing damage to the xylem. Once a window was created, it was firmly secured under a stereo microscope (Axio Zoom.V16, Zeiss, Jena, Germany) using duct tape to ensure no movement of the sample while drying. A thin layer of hydrogel (Tensive Gel, Parker Laboratories, Fairfield, NJ, USA) was applied to the exposed xylem surface to improve light transmission and to reduce evaporation from the surface (Brodribb et al. 2017).

For the leaves, we used both the same stereo microscope cited above and optical clamps (for more details, see http://www.opensourceov.org/) (Brodribb et al. 2016). A healthy, mature and undamaged leaf was selected from each branch and fixed with duct tape under the stereo microscope or in optical clamps. During the imaging process, the leaf remained attached to the branch. The leaf surface area (1 cm^2^) was imaged while the leaf progressively dried.

For both leaf and wood tissue, images were taken every 5 min, and the water potential was monitored in bagged leaves with the pressure chamber mentioned above. For measurements of the leaf water potential, we used similar sized leaves that were on the same branch of the imaged leaf, and exposed to similar conditions. The water potential between each measurement interval was estimated assuming a linear decrease during dehydration (Pereira et al. 2020*a*). Images were processed using ImageJ and the ‘OpenSourceOV ImageJ Toolbox’ was used to analyze the images obtained. Embolism events were determined by analyzing differences in pixels of individual images from the previous images due to changes in the brightness of the xylem, which were then transformed into masks for quantification. The percentage of embolized pixels (PEP) was quantified, while the sample dehydrated over time (Brodribb et al. 2016, 2017), and Ψ_50_, Ψ_12_ and Ψ_88_ were calculated using Eqs (4) to (6).

A Pneumatron was connected to a *Citrus* branch, while either a leaf or a branch from the same terminal branch was selected for optical imaging. For *A. pseudoplatanus*, *C. betulus*, *F. sylvatica*, *P. avium*, *Q. petraea* and *Q. robur*, we used optical data from Guan et al. (2022). Guan and colleagues collected samples under similar conditions from trees at Ulm University, and in the same forest from which we took some of our samples for pneumatic VCs. Therefore, we were able to compare Ψ_12_, Ψ_50_ and Ψ_88_ values obtained with the Pneumatic method with those obtained by Guan et al. (2022) based on the optical method. Comparison with the optical method was chosen because both the optical and pneumatic methods provide continuous and automated data, while other standard methods such as the bench dehydration method and the flow-centrifuge quantify the loss of hydraulic conductivity at selected intervals during dehydration.

The xylem water potential at turgor loss point (Ψ_TLP_) was based on literature data for *A. campestre*, *A. pseudoplatanus*, *C. betulus*, *C. sinensis*, *C. arabica*, *E. camaldulensis*, *F. sylvatica*, *O. europaea*, *P. tremula*, *P. avium*, *Q. petraea* and *Q. robur* (Table S1 available as Supplementary data at *Tree Physiology* Online).

The difference between Ψ_12_, Ψ_50_ and Ψ_88_ derived from the pneumatic VCs (with and without the adjustment) and the optical method was quantified as the root-mean-squared deviation (RMSD)


(7) \begin{equation*} \mathrm{RMSD}=\sqrt{\frac{1}{n-1}\ {\sum}^n{\left({\Psi}_x OV-{\Psi}_x\mathrm{Pneumatron}\right)}^2}, \end{equation*}

where Ψ_x_ means Ψ_12_, Ψ_50_ and Ψ_88_ (in MPa) obtained with the optical method (OV) or with the Pneumatron.

### Sap extraction from cut-open vessels

This experiment was made to check if sap could be extracted from cut-open vessels of branches under negative pressure. Here, we used the same plants of *A. pseudoplatanus*, *C. sinensis*, *C. arabica*, *F. sylvatica*, *P. avium*, *Q. robur* and *Q. petraea*. For *E. camaldulensis* and *O. europaea*, 5-year-old saplings were used since the trees used for the VCs in Brazil were not available in Germany. Branches were cut from plants early in the morning and transferred into plastic bags to the laboratory. The plants were left inside the plastic bags for at least 2 h before the measurements of water potential and then sap was extracted. One branch was used for each measurement. The branches were left to dehydrate on a bench for varying periods of time to obtain several water potentials and establish the association between dehydration level and sap extracted. Xylem water potential varied from −0.1 to −8 MPa and was measured using a pressure chamber based on a leaf that was bagged in a dark plastic bag for 1 h to minimize transpiration. After that, the terminal part of the branch was cut off, leaving a leafless segment. The length of this branch segment was about half the mean vessel length for each species to assure that a large number of vessels were cut open at both sides. Mean vessel lengths were obtained from literature or measured as described below (Table S2 available as Supplementary data at *Tree Physiology* Online).

Immediately after cutting, the proximal end of the branch segment was connected to a Pneumatron apparatus and a vacuum reservoir of 100 mL using elastic tubing and plastic clamps. We did not debark the stem end connected to a Pneumatron as we wanted to replicate the standard Pneumatron protocol without bark removal. The distal end was kept exposed to the atmosphere. Inside the elastic tubing, there was an 8-mm cigarette filter (Zig-Zag, Slim filter, 6 mm, Spain), which had been weighed (W_1_) accurately with a balance. One cotton filter was used for each measurement. The cotton was in direct contact with the sample to collect exudated sap. A vacuum of about 40 kPa was applied for 2 min to the sample to ensure that all sap would be extracted (Pereira et al. 2020*b*), and then, the cotton filter was weighed again (W_2_). Then, branch dry mass (*B*_dry_) was determined after drying samples in an oven with forced air circulation at 60 °C until constant weight. We assumed that the weight difference between cotton filters before and after the vacuum (W_1_ and W_2_, respectively) was due to sap extracted from cut-open vessels. Since not all branch segments had a similar size, we normalized the extracted sap by the dry mass of each sample


(8) \begin{equation*} \mathrm{SAP}=\frac{\left({W}_1-{W}_2\right)}{B_{\mathrm{dry}}}, \end{equation*}

where SAP is the mass of sap extracted from samples with cut open vessels, which was standardized by the sample dry mass (*B*_dry_). The SAP was thus expressed in mg of extracted sap per g of dry mass. The measurements ended when the amount of sap extracted reduced by 99% from the maximum value measured.

### Mean vessel length measurements

For *C. sinensis*, *C. arabica* and *O. europaea*, the vessel length distribution was performed with a Pneumatron (Pereira et al. 2020*b*; Peng et al. 2022). For this purpose, measurements of gas conductivity were made after successive shortening the branches. The length of the cut branch segment was measured with a digital caliper. As the number of open vessels increased with reduced stem length, a higher volume of air could be sucked by the Pneumatron. Vessel length distribution curve and the estimation of mean vessel length were based on Cohen et al. (2003). For *A. pseudoplatanus*, *E. camaldulensis*, *F. sylvatica*, *P. avium*, *Q. petraea* and *Q. robur*, the mean vessel length was based on literature data and was also obtained with the Pneumatron method on similar samples (Table S2 available as Supplementary data at *Tree Physiology* Online, Pereira et al. 2020*b*; Guan et al. 2022).

### Data analyses

Data processing and statistical analyses were performed using R v.4.3.2 (R Core Team 2023), OriginPro v.9.3 (OriginLab Corporation, Northampton MA, USA), and JASP software (https://jasp-stats.org). Spearman nonparametric test was used to assess if the relationship between sap extracted and water potential could be described using a monotonic function. Values of Ψ_12_, Ψ_50_ and Ψ_88_ were compared using Bayesian statistics. After the detection of significant effects in Bayesian ANOVA, Bayes Factors (BF_10_) were used to compare mean values. Our interpretation of Bayes Factors as evidence for an alternative hypothesis (H_1_) was based on Raftery (1995): 1 < BF_10_ < 3 indicated weak support for H_1_; 3 < BF_10_ < 20 indicated positive support for H_1_; and BF_10_ > 20 indicated strong support for the alternative hypothesis.

## Results

### Embolism VCs of Citrus branches and leaves

**Figure 2:**
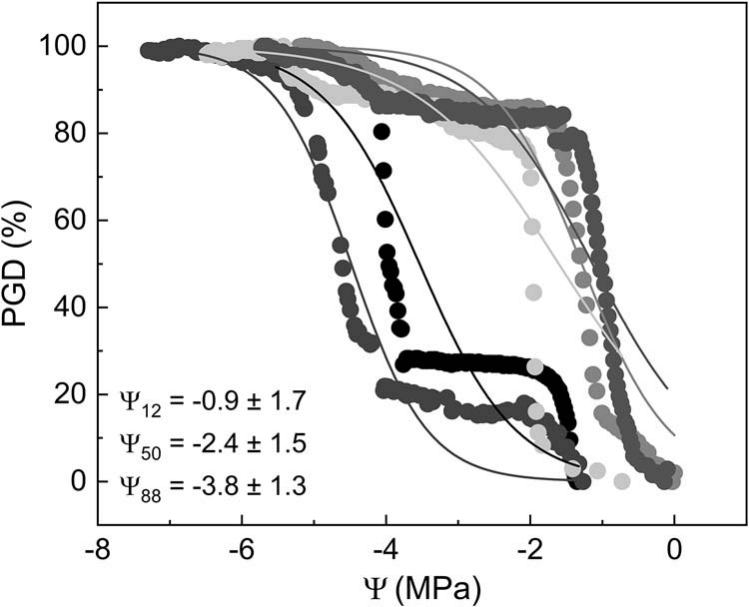
Embolism VCs of *Citrus* branches obtained with a Pneumatron showing five replicates with varying curve shapes. Lines represent the logistic function by Pammenter and Vander Willigen (1998). Ψ_12_, Ψ_50_ and Ψ_88_ mean values ± standard deviation are shown. PGD stands for the percentage gas discharge extracted with a Pneumatron, and Ψ is the xylem water potential.

When constructing pneumatic VCs of *Citrus* plants, some curves were exponential, with Ψ_50_ close to −1 MPa, and even positive values of Ψ_12_ were obtained (Fig. 2). A large discrepancy between the curves and therefore between Ψ_12_ and Ψ_50_ values was found (Fig. 2). Mean values of Ψ_12_ and Ψ_50_ were −0.9 MPa and −2.4 MPa, with a standard deviation of 1.7 and 1.5 MPa, respectively.

**Figure 3:**
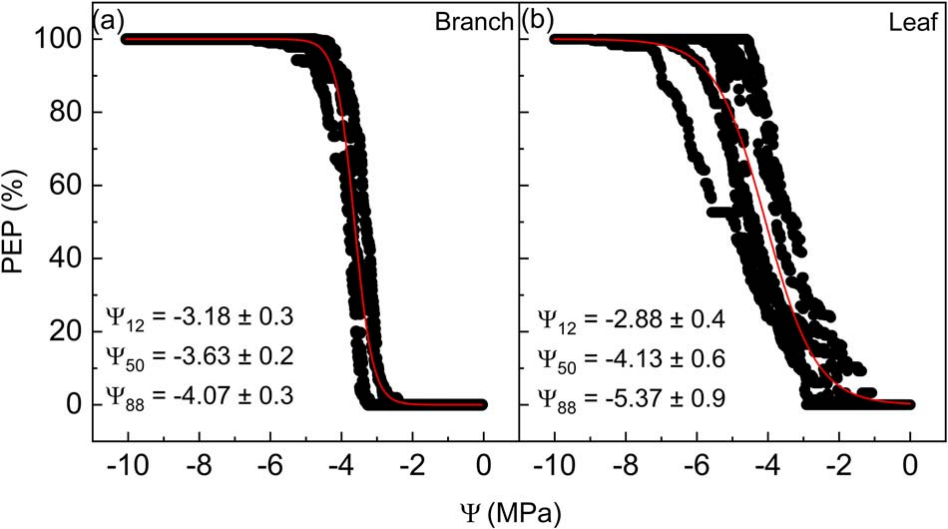
Embolism VCs of *citrus* plants using the optical method for branches (a, *n* = 4) and leaves (b, *n* = 8). Red lines represent the logistic function by Pammenter and Vander Willigen (1998). Ψ_12_, Ψ_50_ and Ψ_88_ values (MPa) are presented, mean ± standard deviation. PEP stands for the percentage of embolized pixels, and Ψ is the xylem water potential.

Embolism resistance of leaf and branch xylem based on the optical technique revealed mean Ψ_50_ of −3.63 ± 0.2 and −4.13 ± 0.6 MPa for branches and leaves, respectively (Fig. 3). There was no statistical difference for Ψ_12_ and Ψ_50_ between leaves and branches using the optical method, while Ψ_88_ was less negative for branches (BF_10_ = 3.2).

### Sap extracted from cut-open vessels

**Figure 4:**
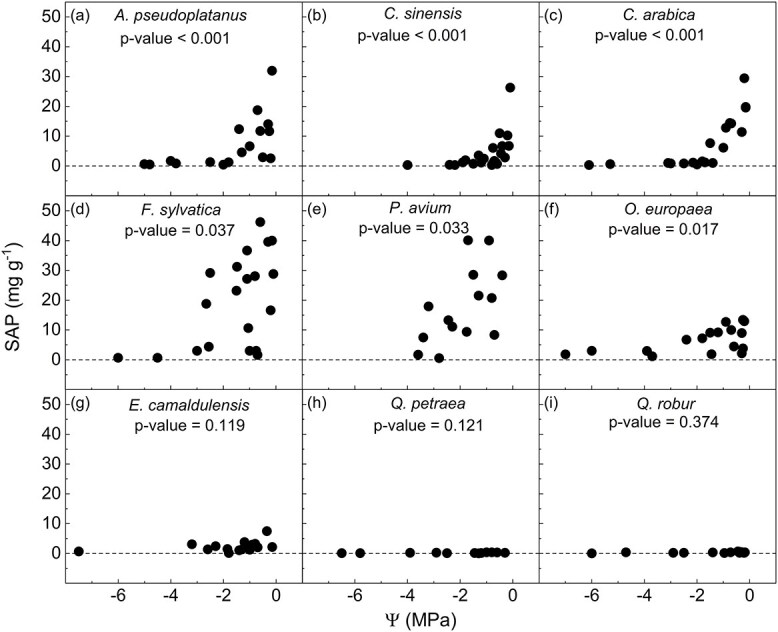
Relationship between the weight of SAP extracted from cut-open vessels (SAP in mg of extracted SAP per g of branch dry weight) and xylem water potential (Ψ) measured during bench dehydration for *A. pseudoplatanus* (a, *n =* 17), *C. sinensis* (b, *n =* 24), *C. arabica* (c, *n =* 19), *F. sylvatica* (d, *n =* 20), *P. avium* (e, *n =* 14), *O. europaea* (f, *n =* 17), *E. camaldulensis* (g, *n =* 15), *Q. petraea* (h, *n =* 12) and *Q. robur* (i, *n =* 14). Each symbol represents one measurement. The *P*-values of the Spearman correlation test are shown.

Sap was extracted from cut-open conduits in six out of nine species tested. There was a significant correlation (*P* < 0.05) between the amount of sap extracted and xylem water potential for *A. pseudoplatanus*, *C. sinensis*, *C. arabica*, *F. sylvatica*, *O. europaea* and *P. avium* (Fig. 4a–f), and xylem sap residue became less likely with decreasing xylem water potential. The amount of residual sap differed among the species, and *O. europaea*, for instance, showed a low amount of sap extracted (Fig. 4f). For *E. camaldulensis*, *Q. robur* and *Q. petraea*, almost no sap was extracted, and the amount of sap was fairly constant across a wide range of xylem water potentials (*P* > 0.05, Fig. 4g–i).

### Adjustment of VCs by considering the stability of the initial gas discharge plateau

**Figure 5:**
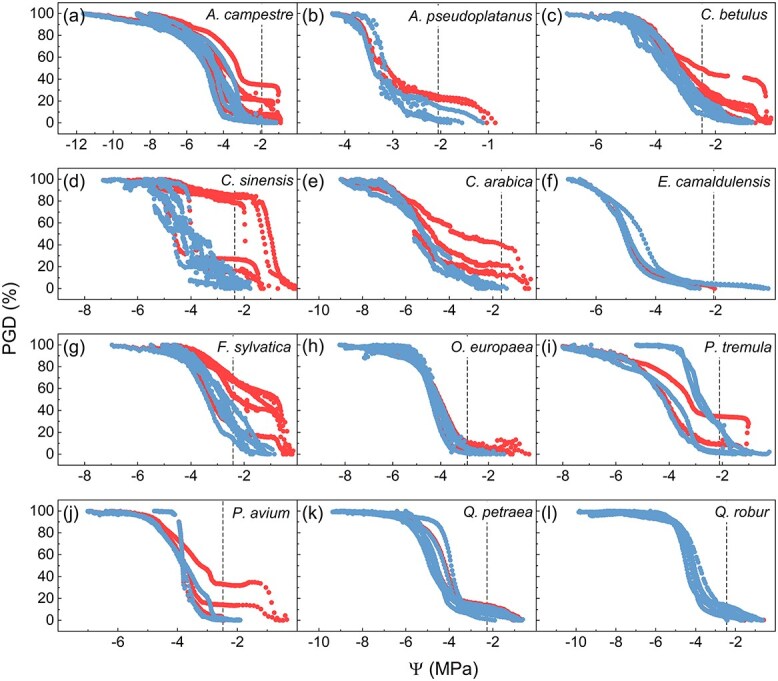
Embolism VCs obtained with a Pneumatron without (red symbols) and with (blue symbols) correction for xylem sap residue in cut-open conduits for *A. campestre* (a, *n =* 6), *A. pseudoplatanus* (b, *n =* 3), *C. betulus* (c, *n =* 6), *C. sinensis* (d, *n =* 5), *C. arabica* (e, *n =* 3), *E. camaldulensis* (f, *n =* 3), *F. sylvatica* (g, *n =* 6), *O. europaea* (h, *n =* 5), *P. tremula* (i, *n =* 4), *P. avium* (j, *n =* 3), *Q. petraea* (k, *n =* 6) and *Q. robur* (l, *n =* 6). Dashed lines indicate the water potential at the turgor loss point (Ψ_TLP_). PGD stands for the percentage gas discharge and Ψ is the xylem water potential. For a list of Ψ_50_ values, see Table S2 available as Supplementary data at *Tree Physiology* Online.

When analyzing pneumatic VCs of various species from different environments, there was an increased amount of gas discharge in several but not all species. The VCs were adjusted by using the method described in Materials and methods section, and then original and adjusted VCs were compared with each other (Fig. 5). In all adjusted VCs, the initial plateau was clearly identified before the turgor loss point had been reached (Ψ_TLP_, Table S1 available as Supplementary data at *Tree Physiology* Online), as shown by the dashed lines in Fig. 5. In 3 of the 12 species studied (*E. camaldulensis*, *Q. petraea* and *Q. robur*), there was no need for adjustment, because the VCs showed a stable plateau during the first stages of sample dehydration, and thus, no initial shift in the amount of gas extracted (Fig. 5). For *O. europaea*, a small shift in GD was detected under high xylem water potential (Fig. 4f). For the other eight species (*A. campestre*, *A. pseudoplatanus*, *C. betulus*, *C. sinensis*, *C. arabica*, *F. sylvatica*, *P. tremula* and *P. avium*), an early shift in GD was detected (Fig. 5).

**Figure 6:**
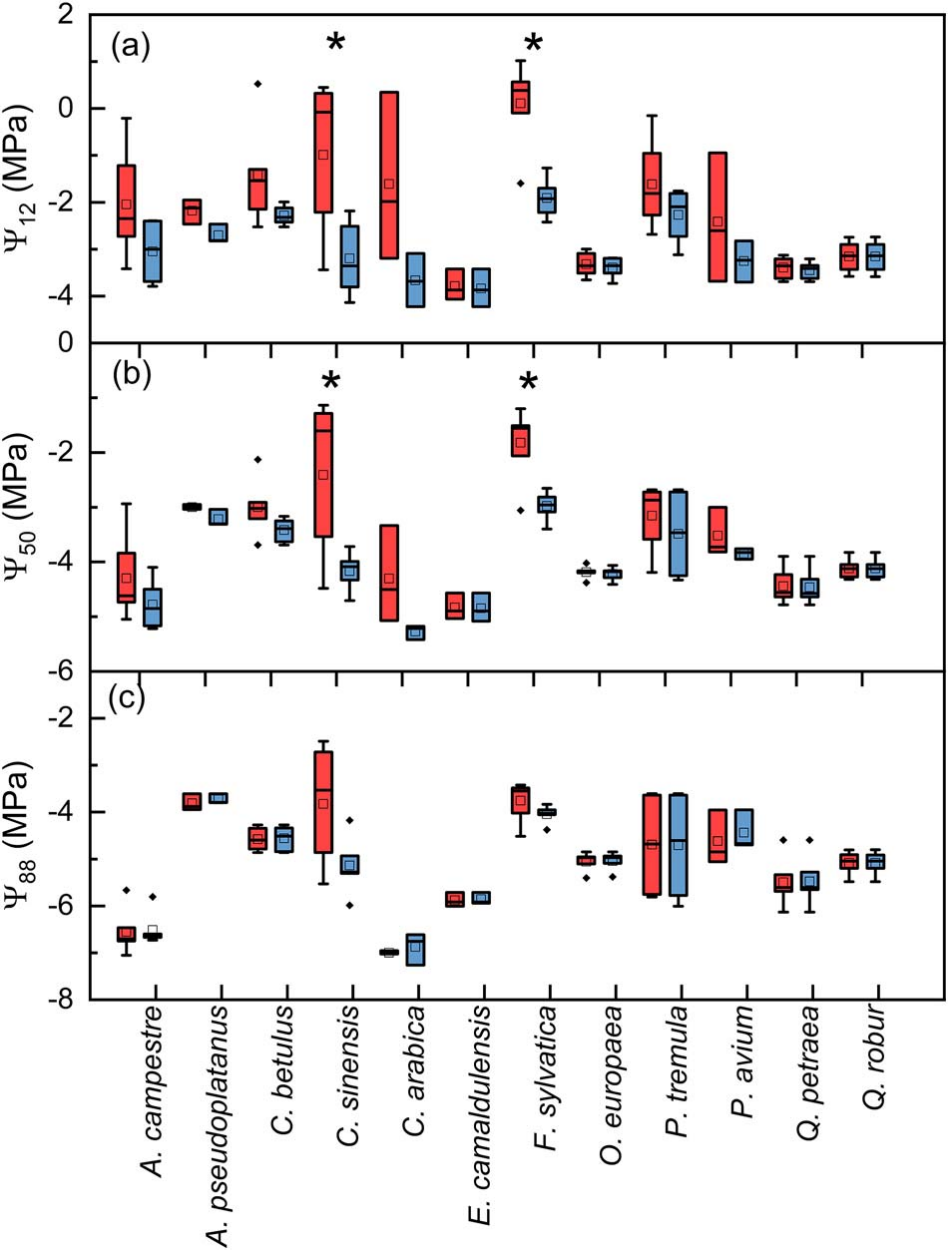
Values of Ψ_12_ (in a), Ψ_50_ (in b) and Ψ_88_ (in c) derived from VCs obtained with the Pneumatron without (red) and with (blue) correction for xylem sap residue in cut-open conduits. Boxplots consider the median, 25th and 75th percentiles, and mean values are represented by squares (*n* = 3 to 6). ^*^Indicates differences between adjusted and nonadjusted values for a given species. Filled diamonds indicate outliers that were determined as 3 ^*^ IQR > outliers > 1.5 ^*^ IQR, where IQR is the interquartile range.

Statistical differences for Ψ_12_ and Ψ_50_ with and without the correction were found for *C. sinensis* (BF_10_ = 2.8 and 2.5, respectively) and *F. sylvatica* (BF_10_ = 43 and 13, respectively) (Fig. 6a and b). Besides, Ψ_88_ was not significantly affected by the initial plateau adjustment in any species. Although we found changes in Ψ_12_ when comparing VCs with adjusted and nonadjusted plateaus for *A. campestre*, *A. pseudoplatanus*, *C. betulus*, *C. arabica*, *P. tremula* and *P. avium*, there was no statistical difference for this parameter probably due to a large standard deviation found without the adjustment (Fig. 6; Table S3 available as Supplementary data at *Tree Physiology* Online). In fact, the standard deviations of all parameters derived from VCs were considerably reduced when we applied the correction for the artifact caused by residual xylem sap (Figs 5 and 6; Table S3 available as Supplementary data at *Tree Physiology* Online).

**Figure 7:**
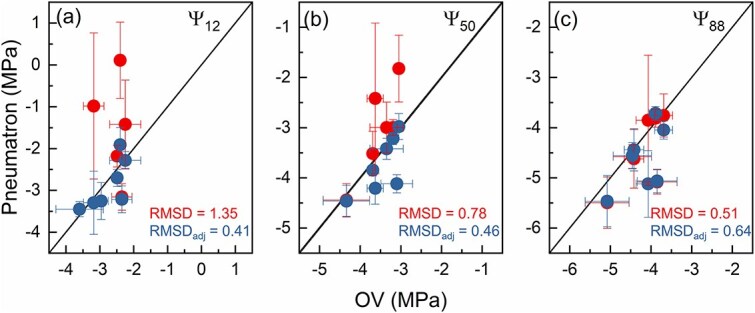
Correlations between Ψ_12_ (a), Ψ_50_ (b) and Ψ_88_ (c) obtained with the optical method (OV) and the Pneumatron method for *A. pseudoplatanus*, *C. betulus*, *C. sinensis*, *F. sylvatica*, *P. avium*, *Q. petraea* and *Q. robur*. Values are presented as mean ± standard deviation. Pneumatron data were adjusted (blue) or nonadjusted (red). The OV data for *A. pseudoplatanus*, *C. betulus*, *F. sylvatica*, *P. avium*, *Q. petraea* and *Q. robur* were taken from Guan et al. (2022). For *C. sinensis*, the OV data were measured and shown in Fig. 3. Values at the bottom represent the RMSD in MPa for nonadjusted curves (red) and adjusted curves (blue).

When comparing Ψ_12_, Ψ_50_ and Ψ_88_ values obtained with the Pneumatic method with those obtained with the optical method (Guan et al. 2022), a large discrepancy was observed for the pneumatic Ψ_12_ when no initial plateau adjustment was applied (red symbols, Fig. 7a): Ψ_12_ values were considerably higher for the pneumatic than the optical method. However, the discrepancy for Ψ_12_ and Ψ_50_ between the methods was considerably reduced when a correction of the pneumatic VCs was made. With the correction, the RMSD reduced from 1.35 to 0.41 MPa for Ψ_12_, and from 0.78 to 0.46 MPa for Ψ_50_ (blue symbols, Fig. 7a and b). For Ψ_88_, the RMSD increased slightly from 0.51 to 0.64 MPa after the adjustment, which is a minor increase compared with the reduction observed for Ψ_12_ (Fig. 7c).

## Discussion

Our results show that an increase in gas discharge during the initial stages of xylem dehydration occurred in 8 out of 12 species, although it only affected significantly the pneumatic Ψ_12_ and Ψ_50_ estimation for *C. sinensis* and *F. sylvatica* (Fig. 6). However, no effect was found for the estimation of Ψ_88_ for all species. Our analysis of the slopes of the pneumatic VCs allowed us to detect and correct an artificial increase in gas discharge and improve our estimations of embolism resistance. The evidence for supporting the applied correction is that the pneumatically corrected VCs were largely similar to the VCs obtained with the optical method (Fig. 7). As expected, the absolute effect of the correction was more pronounced for Ψ_12_ than for Ψ_50_ and Ψ_88_ (Figs 5 and 6). Besides, the standard deviation was considerably reduced when the stability of the initial plateau in VCs was corrected. The standard deviations for Ψ_12_ and Ψ_50_ of *Citrus* plants, for instance, were reduced by 53% and 80%, respectively (Fig. 6; Table S3 available as Supplementary data at *Tree Physiology* Online).

The shift in GD during initial dehydration stages was found in eight species, and we noticed minimal differences when comparing original and adjusted Ψ_50_ values for 10 out of the 12 species studied. The large intraspecific variability in whether or not this artifact occurs may explain why some previous methodological comparisons on embolism resistance did not detect the artifact reported here (Zhang et al. 2018; Sergent et al. 2020; Pereira et al. 2020*a*; Guan et al. 2021; Paligi et al. 2023). Our findings may also explain why previous reports described a weak correlation for Ψ_12_ estimated with various methods (Fig. 7; Yang et al. 2023; Paligi et al. 2023). As shown in Fig. 7a, the proposed adjustment can improve the accuracy of the Ψ_12_ estimation, at least when comparing the Pneumatic and optical methods. In addition, our approach to focus on temporal changes in the slope of VCs also addresses the controversy on the subjective detection of the initial and final plateaus (Brum et al. 2023; Chen et al. 2023). In fact, these plateaus can be mathematically detected. Similar to the initial plateau, the final plateau was unproblematic for the species studied here.

Our experiments showed that not all xylem sap in cut-open conduits may be removed when cutting well-hydrated samples in air. This observation provides an interesting explanation for changes in the gas discharge amount that do not reflect embolism propagation in intact conduits. Our measurements revealed that the amount of sap extracted from cut-open vessels was related to the xylem water potential in *A. pseudoplatanus*, *C. sinensis*, *C. arabica*, *F. sylvatica* and *P. avium* (Fig. 4a–e). In VCs of those species, we noticed an increase in gas discharge during the initial dehydration (Fig. 5b, d, e, g and j). In another group of species (*E. camaldulensis*, *Q. robur* and *Q. petraea*), almost no sap was extracted in cut samples, regardless of the water potential (Fig. 4). Interestingly, VCs of *E. camaldulensis*, *Q. robur* and *Q. petraea* did not show any increase in gas discharge under high water potentials (Fig. 5).

**Figure 8:**
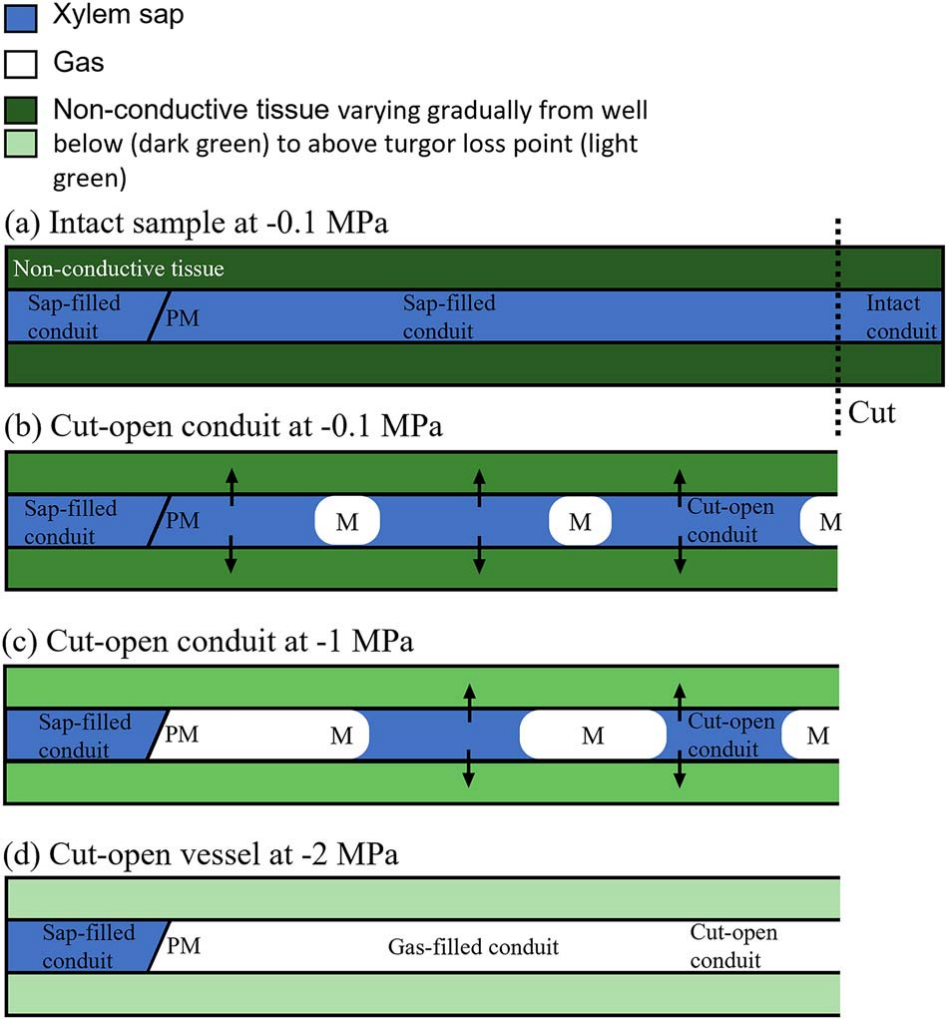
Gas–liquid dynamics in a hypothetical branch with a turgor loss point of −2 MPa. (a) Hydrated intact branch, before cutting it at −0.1 MPa, with sap-filled conduits. (b) The same branch at high water potential after cutting, some menisci are formed as large gas bubbles, and water starts to be drained to rehydrate the surrounding tissue. As this tissue still has turgor (given by the dark green color in surrounding, nonconductive tissue), not all sap is drained immediately. (c) More xylem sap is absorbed by the nonconductive cells as surrounding tissues dehydrate further. (d) Complete uptake of xylem sap by nonconductive cells, with the cut-open conduit being completely filled with air, which happens at a water potential close to the turgor loss point. Arrows indicate sap movement. PM = intervessel wall with pit membranes, and M = meniscus.

According to the cohesion-tension theory, xylem sap is under subatmospheric to negative pressures when plants are undergoing transpiration. Thus, xylem sap should be drained from cut-open vessels to rehydrate the surrounding tissue, and this would happen immediately when cutting samples in air (Dixon and Joly 1895; Tyree et al. 2003). As such, the observation that residual sap is left in cut-open conduits requires an explanation. We speculate that xylem sap may be not quickly removed from cut-open conduits when plants are fairly well-hydrated. When applying a partial vacuum with the Pneumatron to short segments, the amount of sap extracted from fully cut-open conduits depended on the xylem water potential, and this was especially the case for species that showed an initial increase in gas discharge (Fig. 4a–f). The highest amount of sap was extracted under well-hydrated conditions for *A. pseudoplatanus*, *C. sinensis*, *C. arabica*, *F. sylvatica* and *P. avium*. On the other hand, we could not extract sap from species with no initial increase in gas discharge (Fig. 4f–h). If xylem water potentials are close to zero, it is possible that, after cutting xylem tissue in air, the sap is only gradually drained by the surrounding tissue. As long as this surrounding tissue, which may include axial and ray parenchyma cells and living fibers, is under turgor pressure larger than one atmosphere, xylem sap may not be drained immediately after cutting fresh samples (Fig. 8b). Why some species show the artifact more pronounced than others is unclear, but we speculate that variation in tissue fractions and connectivity of vessels within living cell types could be an explanation.

Vessels in angiosperms are commonly surrounded by highly specialized living parenchyma cells. These vessel-associated cells can be connected to vessels via half-bordered pits (Morris et al. 2018). Interestingly, the influx of water from vessel-associated parenchyma cells has been observed based on microCT imaging and also proposed as a mechanism to refill embolized vessels (Brodersen et al. 2010, 2018). Knipfer et al. (2016), for instance, found that water droplets emerge from the inner vessel walls adjacent to the xylem parenchyma or fibers in excised branches. Thus, an alternative explanation for the sap extracted from branches with cut-open vessels in our study could be the active secretion of liquid from living cells that are directly connected to conduits. For the moment, we cannot exclude this hypothesis. Vessel-associated parenchyma cells are also related to tylosis formation and gel production, where balloon-like sacs of cytoplasm enter the embolized vessel resulting in partial or full vessel occlusion (Morris et al. 2018). Vessel occlusion by tyloses would strongly affect gas extraction permanently. However, we can exclude this hypothesis, because the artifact described only occurred during initial dehydration stages and was not permanent.

Since it is unclear how fast xylem sap is withdrawn from cut-open conduits in a particular sample, we suggest that removing all data before an initial plateau in GD has been achieved as a general protocol for pneumatic VCs, and especially if an initial increase in GD is detected under high water potential. This procedure can be useful to reduce the variability in embolism resistance, even if the artificial increase in GD is small. For instance, applying a correction to data obtained for *C. arabica* reduced the standard deviation from 1.8 to 0.6 MPa, and from 0.9 to 0.1 MPa for Ψ_12_ and Ψ_50_, respectively (Fig. 6; Table S3 available as Supplementary data at *Tree Physiology* Online). Removing GD measurements before an initial plateau has been reached is a reasonable approach for improving the estimation of Ψ_12_ and Ψ_50_ in some species, since xylem sap appears to be completely absorbed when the surrounding tissues lose turgor. Yet, Pneumatron users will not be required to accurately measure Ψ_TLP_, because a stable plateau in GD should be obtained before the turgor loss point has been reached. The removal of all points before the initial plateau in pneumatic VCs would still be a safe strategy since embolism formation is unlikely before plants lose cell turgor. Previous studies have shown that stomata close at or before turgor loss, earlier than the onset of embolism (Brodribb and Holbrook 2003; Nardini et al. 2003; Brodribb et al. 2016; Skelton et al. 2018). In addition, Martin-StPaul et al. (2017) found that most species close their stomata at water potentials before embolism starts.

A practical consequence of our results is that VCs obtained with the Pneumatic method can be started with slightly dehydrated branches, at least up to **Ψ**_**TLP**_, which facilitates branch sampling when it is not possible to find a completely hydrated plant in the field, or when plants are already transpiring during the day. Starting with slightly dehydrated samples and not putting them under water after cutting would help the gas–liquid meniscus to pull back more quickly, which would especially be appropriate for drought-resistant species with fairly high embolism resistance, such as *Citrus* species.

## Conclusion

For the first time, we report the presence of xylem sap in cut-open vessels affecting embolism VCs obtained with the Pneumatic method. As an easy correction for improving the estimation of important parameters related to embolism resistance, we propose the removal of all points before the initial plateau in pneumatic VCs if an initial shift in GD is detected based on a temporal analysis of the VC slope values. As long as a Pneumatron with high temporal resolution is used, detection of a stable plateau before embolism occurs is feasible.

## Supplementary Material

Supplementary_material2_final_tpae092

## Data Availability

The data supporting the findings of this study are available within the paper and supplementary files.
