# Supplementary material for: Xylem sap residue in cut-open conduits can affect gas discharge in pneumatic experiments
Source: Tree Physiol. 2024 Jul 24;46(13):70–80. doi: 10.1093/treephys/tpae092 (PMC13016742; doi:10.1093/treephys/tpae092)
Supplement: Supplementary_material2_final_tpae092 [file supplementary_material2_final_tpae092.docx]

**Supplementary Data**

**Table S1.** The osmotic potential at turgor loss point (**Ψ_TLP_,** MPa) for the species studied.

| Species | Ψ_TLP_  (mean and range) | References |  |
| --- | --- | --- | --- |
| *Acer campestre* | -1.92 ± 0.40  (-1.28 to -2.51) | Burghardt and Riederer 2003, Nardini et al. 2012, Banks et al. 2019, Kunert and Tomaskova 2020, Thom et al. 2022 |  |
| *Acer pseudoplatanus* | -2.05 ± 0.42  (-1.40 to -2.75) | Nardini et al. 2012, Sjöman et al. 2015, Li et al. 2016, Lübbe et al. 2017, Leuschner et al. 2019, Kunert and Tomaskova 2020 |  |
| *Carpinus betulus* | -2.45 ± 0.35  (-1.85 to -2.71) | Li et al. 2016, Lübbe et al. 2017, Leuschner et al. 2019, Kunert and Tomaskova 2020 |  |
| *Citrus sinensis* | -2.31 ± 0.49  (-1.69 to -2.82) | Savé et al. 1995, Gonçalves et al. 2016, Silva et al. 2019, Miranda et al. 2022 |  |
| *Coffea arabica* | -1.60 ± 0.20  (-1.34 to -1.82) | Meinzer et al. 1990, Da Matta et al. 1993, Nardini et al. 2014 |  |
| *Eucalyptus camaldulensis* | -2.06 ± 0.22  (-1.8 to -2.4) | Dreyer et al. 1992, White et al. 2000, Lemcoff et al. 2002, Siddiqui et al. 2008 |  |
| *Fagus sylvatica* | -2.41 ± 0.30  (-1.80 to -2.82) | Aranda et al. 1996, Backes and Leuschner 2000, Leuschner et al. 2001, 2019, Burghardt and Riederer 2003, Lübbe et al. 2017, Tomasella et al. 2018, Kunert and Tomaskova 2020 |  |
| *Olea europaea* | -2.88 ± 0.72  (-1.67 to 3.57) | Hinckley et al. 1980, Dichio et al. 1997, 2003, Bacelar et al. 2006 |  |
| *Populus tremula* | -2.10 | Kunert and Tomaskova 2020 |  |
| *Prunus avium* | -2.48 ± 0.18  (-2.24 to -2.71) | Peschiutta et al. 2013, Kunert and Tomaskova 2020 |  |
| *Quercus petraea* | -2.27 ± 0.45  (-1.3 to -2.77) | Aranda et al. 1996, Backes and Leuschner 2000, Thomas and Gausling 2000, Burghardt and Riederer 2003, Nardini et al. 2012, Kunert and Tomaskova 2020 |  |
| *Quercus robur* | -2.45 ± 0.22  (-2.16 to -2.68) | Thomas and Gausling 2000, Kunert and Tomaskova 2020, Hanley et al. 2021 |  |

**Table S2.** The mean vessel length for the species studied based on pneumatic measurements.

| Species | Mean vessel length (cm) | References |  |
| --- | --- | --- | --- |
| *Acer pseudoplatanus* | 3.98 ± 1.62 | Guan et al. 2022 |  |
| *Citrus sinensis* | 21.0 ± 1.6 | Measured |  |
| *Coffea arabica* | 20.6 ± 2.8 | Measured |  |
| *Eucalyptus camaldulensis* | 27.7 ± 6.4 | Pereira et al. 2020 |  |
| *Fagus sylvatica* | 5.2 ± 0.8 | Guan et al. 2022 |  |
| *Olea europaea* | 14.9 ± 3.4 | Measured |  |
| *Prunus avium* | 10.6 ± 1.7 | Guan et al. 2022 |  |
| *Quercus petraea* | 29.0 ± 7.0 | Guan et al. 2022 |  |
| *Quercus robur* | 23.0 ± 8.9 | Guan et al. 2022 |  |

**Table S3:** Variation of Ψ_12_, Ψ_50_ and Ψ_88_ (in MPa) obtained from VCs obtained with the Pneumatron without adjustment and with adjustment for a residual sap artefact.

| Species | Ψ_12_ | | Ψ_50_ | | Ψ_88_ | |
| --- | --- | --- | --- | --- | --- | --- |
|  | non-adjusted | adjusted | non-adjusted | adjusted | non-adjusted | adjusted |
| *A. campestre* | -2.0 ± 1.1 | -3.1 ± 0.6 | -4.3 ± 0.8 | -4.8 ± 0.4 | -6.6 ± 0.5 | -6.5 ± 0.3 |
| *A. pseudoplatanus* | -2.2 ± 0.3 | -2.9 ± 0.1 | -3.0 ± 0.1 | -3.3 ± 0.1 | -3.8 ± 0.2 | -3.7 ± 0.2 |
| *C. betulus* | -1.4 ± 1.1* | -2.8 ± 0.1 | -3.0 ± 0.5* | -3.7 ± 0.2 | -4.6 ± 0.2 | -4.6 ± 0.3 |
| *C. sinensis* | -1.0 ± 1.7* | -3.4 ± 0.6 | -2.4 ± 1.5* | -4.2 ± 0.3 | -3.8 ± 1.3 | -5.1 ± 0.7 |
| *C. arabica* | -1.6 ± 1.8 | -3.7 ± 0.6 | -4.3 ± 0.9 | -5.3 ± 0.1 | -7.0 ± 0.1 | -6.9 ± 0.3 |
| *E. camaldulensis* | -3.8 ± 0.3 | -3.9 ± 0.3 | -4.8 ± 0.2 | -4.9 ± 0.2 | -5.9 ± 0.2 | -5.8 ± 0.2 |
| *E. uniflora* | 1.1 ± 0.9* | -2.6 ± 0.3 | -0.9 ± 0.4* | -3.1 ± 0.4 | -2.8 ± 0.6 | -3.5 ± 0.6 |
| *F. sylvatica* | 0.1 ± 0.9* | -2.5 ± 0.2 | -1.8 ± 0.7* | -3.4 ± 0.1 | -3.8 ± 0.4 | -4.2 ± 0.2 |
| *O. europaea* | -3.3 ± 0.3 | -3.4 ± 0.2 | -4.2 ± 0.1 | -4.3 ± 0.1 | -5.1 ± 0.2 | -5.0 ± 0.2 |
| *P. tremula* | -1.6 ± 1.1 | -2.6 ± 0.4 | -3.2 ± 0.7 | -3.7 ± 0.7 | -4.7 ± 1.2 | -4.7 ± 1.3 |
| *P. avium* | -2.5 ± 1.1 | -3.1 ± 0.4 | -3.4 ± 0.5 | -3.6 ± 0.5 | -4.2 ± 0.9 | -4.1 ± 0.8 |
| *Q. petraea* | -3.4 ± 0.2 | -3.7 ± 0.3 | -4.4 ± 0.3 | -4.6 ± 0.4 | -5.5 ± 0.5 | -5.4 ± 0.5 |
| *Q. robur* | -3.2 ± 0.3 | -3.5 ± 0.3 | -4.1 ± 0.2 | -4.2 ± 0.2 | -5.1 ± 0.2 | -5.0 ± 0.1 |

**References**

Aranda I, Gil L, Pardos J (1996) Seasonal water relations of three broadleaved species (*Fagus sylvatica* L., *Quercus petraea* (Mattuschka) Liebl. and *Quercus pyrenaica* Willd.) in a mixed stand in the centre of the Iberian Peninsula. Forest Ecology and Management 84:219–229.

Bacelar EA, Santos DL, Moutinho-Pereira JM, Gonçalves BC, Ferreira HF, Correia CM (2006) Immediate responses and adaptative strategies of three olive cultivars under contrasting water availability regimes: Changes on structure and chemical composition of foliage and oxidative damage. Plant Science 170:596–605.

Backes K, Leuschner C (2000) Leaf water relations of competitive *Fagus sylvatica* and *Quercus petraea* trees during 4 years differing in soil drought. Canadian Journal of Forest Research 30:335–346.

Banks JM, Percival GC, Rose G (2019) Variations in seasonal drought tolerance rankings. Trees - Structure and Function 33:1063–1072.

Burghardt M, Riederer M (2003) Ecophysiological relevance of cuticular transpiration of deciduous and evergreen plants in relation to stomatal closure and leaf water potential. Journal of Experimental Botany 54:1941–1949.

Dichio B, Nuzzo V, Xiloyannis C, Celano G, Angelopoulos K (1997) Drought stress-induced variation of pressure-volume relationships in *Olea Europaea* L. cv ‘Coratina’. Acta Horticulturae:401–410.

Dichio B, Xiloyannis C, Angelopoulos K, Nuzzo V, Bufo SA, Celano G (2003) Drought-induced variations of water relations parameters in *Olea europaea*. Plant and Soil 257:381–389.

Dreyer E, Epron D, Matig OY (1992) Photochemical efficiency of photosystem II in rapidly dehydrating leaves of 11 temperate and tropical tree species differing in their tolerance to drought. Annales des Sciences Forestières 49:615–625.

Gonçalves LP, Alves TFO, Martins CPS, de Sousa AO, dos Santos IC, Pirovani CP, Almeida AAF, Filho MAC, Gesteira AS, Soares Filho W dos S, Girardi EA, Costa MGC (2016) Rootstock-induced physiological and biochemical mechanisms of drought tolerance in sweet orange. Acta Physiologiae Plantarum 38

Guan X, Werner J, Cao K ‐F., Pereira L, Kaack L, McAdam SAM, Jansen S (2022) Stem and leaf xylem of angiosperm trees experiences minimal embolism in temperate forests during two consecutive summers with moderate drought. Plant Biol. https://onlinelibrary.wiley.com/doi/10.1111/plb.13384

Hanley PA, Arndt SK, Livesley SJ, Szota C (2021) Relating the climate envelopes of urban tree species to their drought and thermal tolerance. Science of The Total Environment 753:142012.

Hinckley TM, Duhme F, Hinckley AR, Richter H (1980) Water relations of drought hardy shrubs: osmotic potential and stomatal reactivity. Plant, Cell and Environment 3:131–140.

Kunert N, Tomaskova I (2020) Leaf turgor loss point at full hydration for 41 native and introduced tree and shrub species from Central Europe. Journal of Plant Ecology 13:754–756.

Lemcoff JH, Guarnaschelli AB, Garau AM, Prystupa P (2002) Elastic and osmotic adjustments in rooted cuttings of several clones of *Eucalyptus camaldulensis* Dehnh. from southeastern Australia after a drought. Flora 197:134–142.

Leuschner C, Backes K, Hertel D, Schipka F, Schmitt U, Terborg O, Runge M (2001) Drought responses at leaf, stem and fine root levels of competitive *Fagus sylvatica* L. and *Quercus petraea* (Matt.) Liebl. trees in dry and wet years. Forest Ecology and Management 149:33–46.

Leuschner C, Wedde P, Lübbe T (2019) The relation between pressure–volume curve traits and stomatal regulation of water potential in five temperate broadleaf tree species. Annals of Forest Science 76:60.

Li S, Lens F, Espino S, Karimi Z, Klepsch M, Schenk HJ, Schimitt M, Schuldt B, Jansen S (2016) Intervessel pit membrane thickness as a key determinant of embolism resistance in angiosperm xylem. IAWA Journal 37:152–171.

Lübbe T, Schuldt B, Leuschner C (2017) Acclimation of leaf water status and stem hydraulics to drought and tree neighbourhood: alternative strategies among the saplings of five temperate deciduous tree species. Tree Physiology 37:456–468.

Da Matta FM, Maestri M, Barros RS, Regazzi AJ (1993) Water relations of coffee leaves (*Coffea arabica* and *C. canephora*) in response to drought. Journal of Horticultural Science 68:741–746.

Meinzer FC, Grantz DA, Goldstein G, Saliendra NZ (1990) Leaf water relations and maintenance of gas exchange in coffee cultivars grown in drying soil. Plant Physiology 94:1781–1787.

Miranda MT, Espinoza-Núñez E, Silva SF, Pereira L, Hayashi AH, Boscariol-Camargo RL, Carvalho SA, Machado EC, Ribeiro R V. (2022) Water stress signaling and hydraulic traits in three congeneric citrus species under water deficit. Plant Science 319:111255.

Nardini A, Õunapuu-Pikas E, Savi T (2014) When smaller is better: leaf hydraulic conductance and drought vulnerability correlate to leaf size and venation density across four *Coffea arabica* genotypes. Functional Plant Biology 41:972.

Nardini A, Pedà G, Rocca N La (2012) Trade-offs between leaf hydraulic capacity and drought vulnerability: Morpho-anatomical bases, carbon costs and ecological consequences. New Phytologist 196:788–798.

Pereira L, Miranda MT, Pires GS, Pacheco VS, Guan X, Kaack L, Karimi Z, Machado EC, Jansen S, Tyree MT, Ribeiro R V. (2020) A semi-automated method for measuring xylem vessel length distribution. Theor Exp Plant Physiol 32:331–340. https://link.springer.com/10.1007/s40626-020-00189-4

Peschiutta ML, Bucci SJ, Scholz FG, Kowal RF, Goldstein G (2013) Leaf and stem hydraulic traits in relation to growth, water use and fruit yield in *Prunus avium* L. cultivars. Trees - Structure and Function 27:1559–1569.

Savé R, Biel C, Domingo R, Ruiz-Sánchez MC, Torrecillas A (1995) Some physiological and morphological characteristics of citrus plants for drought resistance. Plant Science 110:167–172.

Siddiqui MT, Shah AH, Tariq MA (2008) Effects of fertilization and water stress on *Eucalyptus camaldulensis* seedlings. Journal of Tropical Forest Science 20:205–210.

Silva MC, Sousa ARO, Cruz ES, Schlichting AF, Filho WSS, Gesteira AS, Filho MAC, Costa MGC (2019) Phenotyping of new hybrid citrus rootstocks under water deficit reveals conserved and novel physiological attributes of drought tolerance. Acta Physiologiae Plantarum 41:1–14.

Sjöman H, Hirons AD, Bassuk NL (2015) Urban forest resilience through tree selection-Variation in drought tolerance in *Acer*. Urban Forestry and Urban Greening 14:858–865.

Thom JK, Livesley SJ, Fletcher TD, Farrell C, Arndt SK, Konarska J, Szota C (2022) Selecting tree species with high transpiration and drought avoidance to optimise runoff reduction in passive irrigation systems. Science of the Total Environment 812:151466.

Thomas FM, Gausling T (2000) Morphological and physiological responses of oak seedlings (*Quercus petraea* and *Q. robur*) to moderate drought. Annals of Forest Science 57:325–333.

Tomasella M, Beikircher B, Häberle KH, Hesse B, Kallenbach C, Matyssek R, Mayr S (2018) Acclimation of branch and leaf hydraulics in adult *Fagus sylvatica* and *Picea abies* in a forest through-fall exclusion experiment. Tree Physiology 38:198–211.

White DA, Turner NC, Galbraith JH (2000) Leaf water relations and stomatal behavior of four allopatric Eucalyptus species planted in Mediterranean southwestern Australia. Tree Physiology 20:1157–1165.
